# A commentary on “Lactated Ringer’s solution versus saline fluid resuscitation for reducing progression to moderate-to-severe acute pancreatitis: a systematic review and meta-analysis”

**DOI:** 10.1097/JS9.0000000000003541

**Published:** 2025-09-23

**Authors:** Mingzhu Zhang, Juan Wen

**Affiliations:** a Department of Emergency Intensive Care Medicine, Shanghai Changzheng Hospital, Shanghai, China; b Department of General Surgery, Xiangyang Central Hospital, Affiliated Hospital of Hubei University of Arts and Science, Xiangyang, Hubei, China

*Dear Editor,*

We read with great interest the recent article titled “Lactated Ringer’s solution versus saline fluid resuscitation for reducing progression to moderate-to-severe acute pancreatitis: a systematic review and meta-analysis”^[1]^ published in the International Journal of Surgery. The goal of this study is to clarify how lactated Ringer’s solution (LR) and normal saline (NS) differ in their effects when used as first-line treatments for acute pancreatitis (AP). It offers strong proof that LR can considerably delay the onset of moderate-to-severe pancreatitis. Furthermore, our results show that LR is linked to a shorter total hospital stay, a lower incidence of local complications, and a decreased requirement for intensive care unit admissions, all of which contribute to a better clinical outcome. Future studies must, however, address a number of important concerns.

First, there is a lack of analysis regarding fluid balance and hemodynamic monitoring. Most included studies failed to provide detailed reports on patients’ fluid status, central venous pressure (CVP), or urine output – key hemodynamic parameters for assessing resuscitation efficacy. Without these critical data, it becomes challenging to determine whether fluid resuscitation was adequate or excessive, which significantly impacts interpretation of outcomes. Future research should standardize reporting of fluid intake/loss, CVP, mean arterial pressure (MAP), and urine output. Subgroup analyses should then be conducted based on these parameters to more accurately evaluate the effectiveness and safety of fluid resuscitation strategies.

Second, the study failed to account for patients ‘baseline nutritional status and supportive care approaches. Nutritional support constitutes a crucial component of acute pancreatitis treatment, with early enteral nutrition (EN) particularly shown to improve prognosis. This research did not analyze variations in nutritional support methods across studies or their potential impact on outcomes. To enhance clinical relevance, subgroup analyses should incorporate variables such as type of nutritional support (intravenous vs. enteral), timing of initiation, and energy intake levels. Evaluating these interactions with fluid resuscitation efficacy would significantly improve the study’s clinical applicability.

Third, there is a lack of subgroup analysis for pancreatitis with different etiologies. While the causes of acute pancreatitis (such as biliary, alcoholic, or hyperlipidemic) may influence inflammatory responses and fluid requirements, this study did not conduct etiology-based subgroup analysis, making it impossible to determine whether LRP shows better efficacy in specific patient groups. Future meta-analyses should extract and analyze data from different etiology subgroups to evaluate the therapeutic differences between LRP and NS in patients with various causes, thereby providing evidence for personalized treatment strategies.

This study still has significant limitations in liquid monitoring indicators, nutritional support, etiology stratification, safety assessment, resuscitation timing analysis, and methodological aspects. These limitations affect the depth of the results and their clinical translational value. Future research is recommended to strengthen standardized data reporting, expand safety endpoints, adopt individual patient data meta-analysis, and incorporate multivariate analysis to provide more comprehensive and personalized recommendations for fluid resuscitation strategies^[2,3]^. The TITAN guideline, which demands openness in the reporting of artificial intelligence, was mentioned in connection with all of this^[4]^.

## Data Availability

None.
